# Influence of the In Vitro Culture System on the Preferential Accumulation of Bioactive Compounds in *Stevia rebaudiana* Shoots

**DOI:** 10.3390/plants15152326

**Published:** 2026-07-29

**Authors:** Duli Martínez-Oró, Antonio A. Calderón, María A. Ferrer

**Affiliations:** Department of Agronomic Engineering, Universidad Politécnica de Cartagena Member of European University of Technology EUT+, Paseo Alfonso XIII 48, 30203 Cartagena, Spain; duli.martinez.o@gmail.com (D.M.-O.); mangeles.ferrer@upct.es (M.A.F.)

**Keywords:** *Stevia rebaudiana*, steviol glycosides, phenolic compounds, in vitro culture systems, antioxidant capacity

## Abstract

The shoots of the *Stevia rebaudiana* plant have been identified as a significant source of biologically active compounds. Of these, phenolic compounds and steviol glycosides are of particular significance. The present study explores the effect of diverse in vitro culture systems (i.e., agar-gelled medium, static liquid medium, liquid medium with orbital agitation, and liquid medium with glass beads) on the accumulation of these compounds in stevia shoots. The results demonstrated that the presence of agitated liquid media led to a substantial augmentation in the accumulation of total soluble phenolic compounds (2.4-fold), total soluble hydroxycinnamic acids (1.9-fold), and total soluble flavonoids (1.6-fold) in comparison to shoots cultivated on agar-gelled media (control). The antioxidant capacity determined on shoots exhibited a comparable trend, with increases of 1.9-fold (DPPH assay) and 2.2-fold (FRAP assay) relative to the control. However, the highest accumulation of steviol glycosides was observed in the glass bead system, with increases of 1.4-fold compared to the control and more than 8-fold relative to liquid culture systems. These findings suggest that the biosynthetic pathways leading to phenolics and steviol glycosides are differentially sensitive to the culture system employed, thereby enabling the selection of the stevia shoot cultivation system based on the targeted metabolites.

## 1. Introduction

*Stevia rebaudiana* (Bertoni) Bertoni (Asteraceae) is a perennial plant recognized for its high foliar content of steviol glycosides (SGs), a group of non-caloric diterpenoid compounds with an intense sweetening capacity [1,2]. Among the numerous steviol glycosides (SGs) identified in *S. rebaudiana* [3,4], stevioside (STV) and rebaudioside A (RebA) are generally the most abundant, representing approximately 5–10% and 2–5% of leaf dry weight, respectively [3,5]. Their accumulation, however, is strongly influenced by stevia variety, growth period and environmental conditions [1,6]. Although both compounds contribute substantially to the sweetness of stevia leaves, RebA is generally preferred because of its cleaner taste and reduced bitter aftertaste compared with STV [7]. Consequently, the RebA/STV ratio is a key determinant of the sensory quality of stevia products [8].

Besides their sweetening properties, SGs have been associated with several beneficial physiological effects, including glycaemic regulation, antihypertensive, anti-inflammatory, antioxidant, and anti-obesity activities, with no health risks to consumers [3,4]. However, these health-promoting properties may also be influenced by other bioactive constituents naturally present in *Stevia rebaudiana* leaves, particularly phenolic compounds [9]. The increasing consumer demand for natural ingredients and plant-derived bioactive compounds has reinforced the interest in *Stevia rebaudiana* as a source of high-value sweeteners and health-promoting phytochemicals. However, large-scale production of stevia remains constrained by poor seed viability, low germination rates, prolonged germination, and limited rooting capacity of vegetative cuttings, thereby hindering the large-scale production of uniform planting material [10,11,12,13]. In this sense, micropropagation has become an effective and less time-consuming approach for the clonal propagation of stevia genotypes. Efficient in vitro micropropagation protocols have been established using different explant types, culture media, and combinations of plant growth regulators [10,14,15,16].

In addition, to facilitating rapid clonal propagation, in vitro culture represents a valuable biotechnological tool for providing high-quality planting material for the sustainable production of plant-derived bioactive compounds [17,18]. Organized in vitro cultures (organ cultures) are increasingly recognized as efficient platforms to produce high-value bioactive compounds. Compared with unorganized cultures (cell cultures), they often exhibit greater biosynthetic competence and metabolic stability [19]. However, the success of these approaches largely depends on the plant species, the target metabolites, and the specific culture conditions, which strongly influence plant growth and secondary metabolite accumulation [17,18]. In addition to the composition of the culture medium, its physical state (solid or liquid) is an important factor influencing plant growth and secondary metabolite accumulation in vitro. The different physicochemical properties of solid and liquid culture systems can affect nutrient uptake, water availability, gas exchange and plant physiology, ultimately influencing the biosynthesis of bioactive compounds. Previous studies have reported that liquid culture can increase the accumulation of phenolic compounds in certain species compared to solid media [20,21].

Liquid culture media has been shown to have several advantages over agar-based solid media. One key benefit is the reduction in propagation costs, achieved by eliminating one of the most expensive components of the media: agar [22]. Additionally, it enables the scaling up of cultures and promotes more reproducible responses from plant materials to medium conditions. However, liquid culture media has limitations. Some species are sensitive to excess available water, limited gas exchange, or the loss of polarity in the explants [23]. One effective strategy to leverage the benefits of gel-based and liquid media is to utilize liquid media in conjunction with an inert physical support. In this regard, the literature describes the use of various inert materials in micropropagation assays. Among these, glass beads have demonstrated significant potential for use in micropropagation due to their chemical inertness, uniformity of size and shape, ease of handling, the possibility of nearly indefinite reuse, and their suitability for large-scale culture systems [24]. The utilization of liquid media in conjunction with glass beads as a physical support has yielded superior growth outcomes in comparison to the use of agar-based gel media and liquid media for some species [25], including several medicinal plants [26,27]. However, to the best of our knowledge, there is virtually no information available on its effect on the accumulation of bioactive compounds.

The responses described above are highly species-dependent, and information regarding the influence of different in vitro culture systems on metabolite accumulation in *Stevia rebaudiana* remains limited. To address this gap, four in vitro culture systems (agar-solidified medium, glass bead-supported culture, static liquid culture, and continuous agitated liquid culture) were compared to evaluate their effects on the accumulation of SGs and phenolic compounds.

## 2. Results

### 2.1. Effects of Culture System on Total Antioxidant Capacity

**Figure 1:**
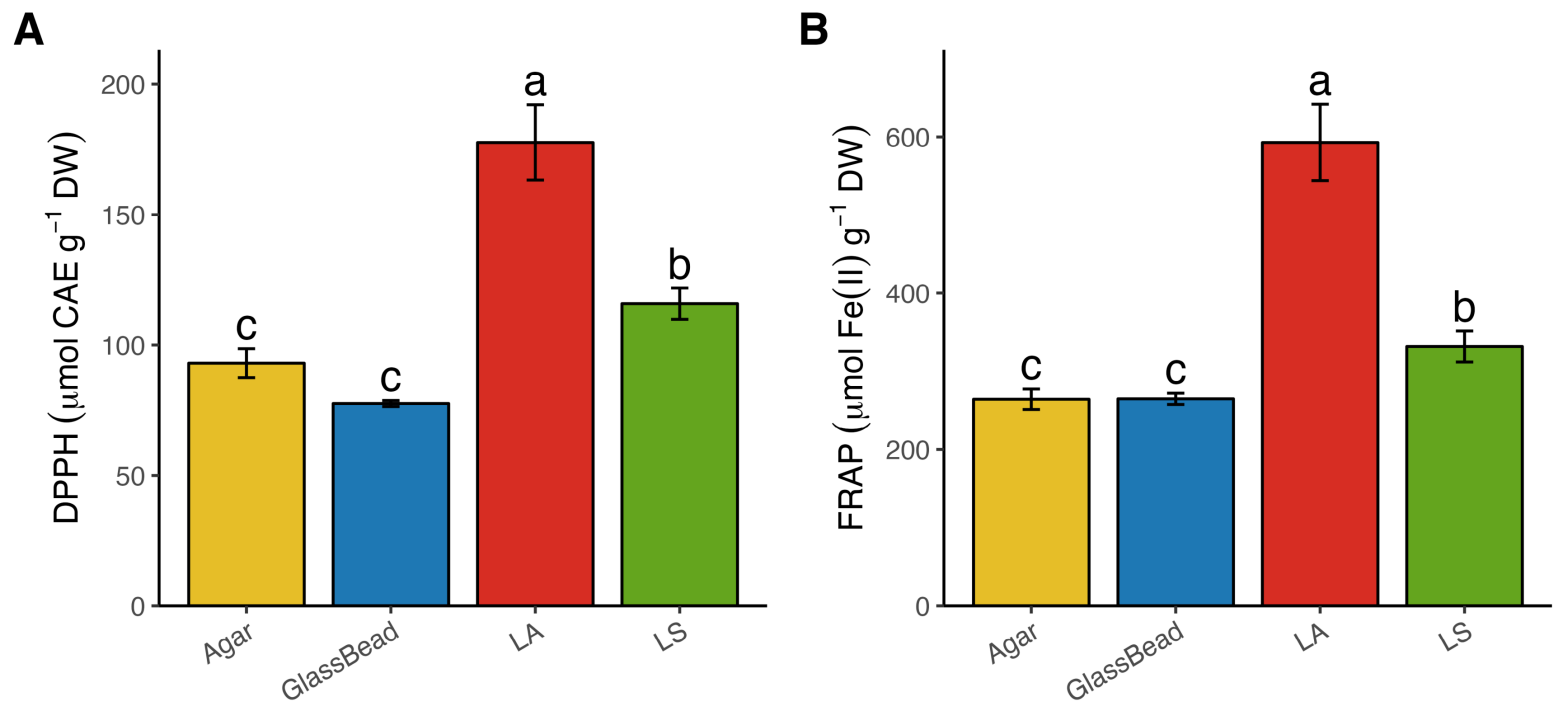
Total antioxidant capacity determined by the DPPH (**A**) and FRAP (**B**) assays in aqueous extracts of four-week-old in vitro-grown stevia shoots cultured under agar-gelled, glass bead-supported, agitated liquid (LA), and static liquid (LS) culture systems. Data represent means ± SD (*n* = 6). Different letters indicate significant differences according to Tukey’s HSD test at *p* < 0.05.

To obtain an overview of the biochemical changes induced by the different culture systems, the total antioxidant capacity (TAC) of stevia shoots was evaluated using the DPPH and FRAP assays. Culture systems significantly affected TAC values in both assays. Shoots grown under agitated liquid culture (LA) exhibited the highest antioxidant capacity, followed by static liquid culture (LS). In contrast, shoots grown on agar-solidified medium (A) and in glass bead-supported cultures showed the lowest TAC values (Figure 1).

### 2.2. Effects of Culture System on Phenolic Compound Accumulation

**Figure 2:**
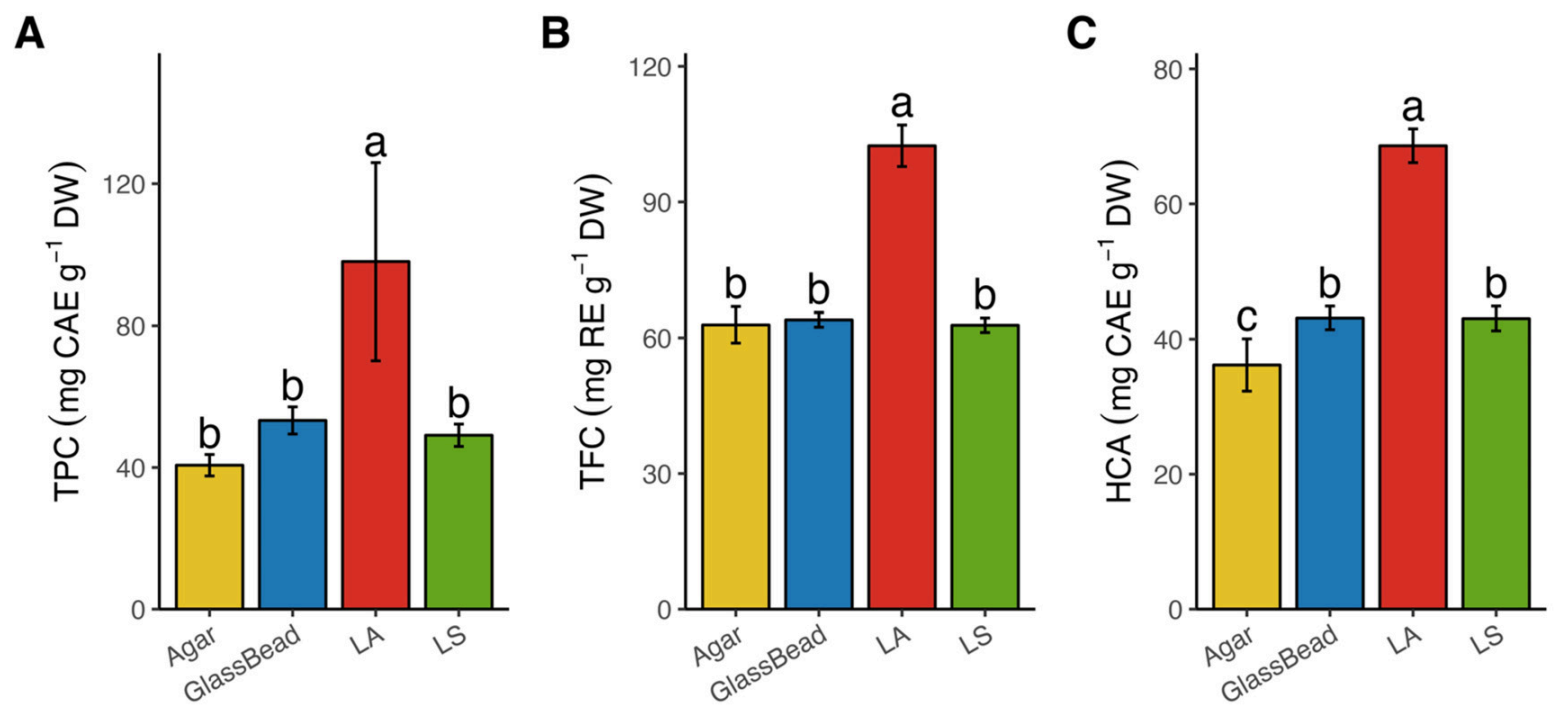
Total soluble phenol content (**A**), flavonoid content (**B**), and hydroxycinnamic acid content (**C**) in aqueous extracts of four-week-old in vitro-grown stevia shoots cultured under agar-gelled, glass bead-supported, agitated liquid (LA), and static liquid (LS) culture systems. Data represent means ± SD (*n* = 6). Different letters indicate significant differences according to Tukey’s HSD test at *p* < 0.05.

To examine whether changes in TAC were associated with phenolic compound accumulation, total soluble phenol content (TPC) was determined (Figure 2A). Shoots cultured under agitated liquid conditions accumulated significantly higher levels of TPC (98 ± 28 mg CAE g^−1^ DW) than those grown under the other culture systems, among which no statistically significant differences were detected.

To further characterize the phenolic subclasses contributing to the enhanced TAC and TPC observed under agitated liquid conditions, the contents of total soluble flavonoids (TFC) and hydroxycinnamic acids (HCAs) were quantified. Consistent with the TPC results, shoots cultured in agitated liquid medium exhibited the highest levels of both TFC (102 ± 5 mg RE g^−1^ DW) and HCAs (69 ± 3 mg CAE g^−1^ DW). No significant differences in TFC were detected among the remaining culture systems (Figure 2B). In contrast, HCA content varied among treatments, with agar-solidified cultures showing the lowest values (Figure 2C).

**Figure 3:**
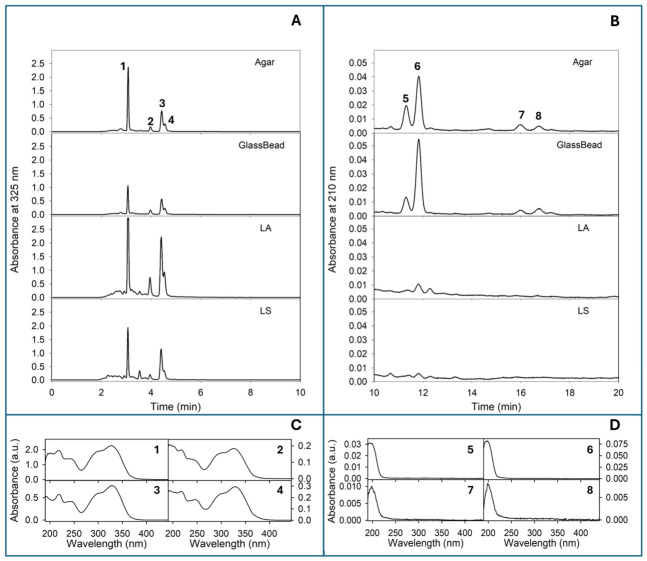
Enlarged regions of representative HPLC chromatograms recorded at 325 nm (**A**) and 210 nm (**B**) of aqueous extracts from in vitro-grown stevia shoots, highlighting the peaks showing the most remarkable differences among culture systems. The corresponding UV-Vis spectra of these peaks are shown below the chromatograms. Peak 1, chlorogenic acid; peak 2, caffeic acid; peaks 3 and 4, unidentified hydroxycinnamic acid derivatives (**C**). Peak 5, rebaudioside A (RebA); peak 6, stevioside (STV); peaks 7 and 8, unidentified steviol glycosides (SGs) (**D**). Complete chromatograms are provided in Supplementary Figure S1.

To determine whether the observed differences in total phenolic content were associated with changes in specific metabolites, HPLC analyses were conducted to profile phenolic compounds as well as the accumulation of steviol glycosides in stevia shoots. As shown in Figure 3, the HPLC chromatograms at 325 nm revealed several compounds with UV/Vis absorption spectra characteristic of hydroxycinnamic acid derivatives. Among these, the peaks 1 and 2 with retention times of approximately 3.1 and 3.9 min, were identified as chlorogenic acid and caffeic acid, along with two additional compounds (peaks 3 and 4) that were present in all extracts and represented major constituents of the phenolic profile.

**Figure 4:**
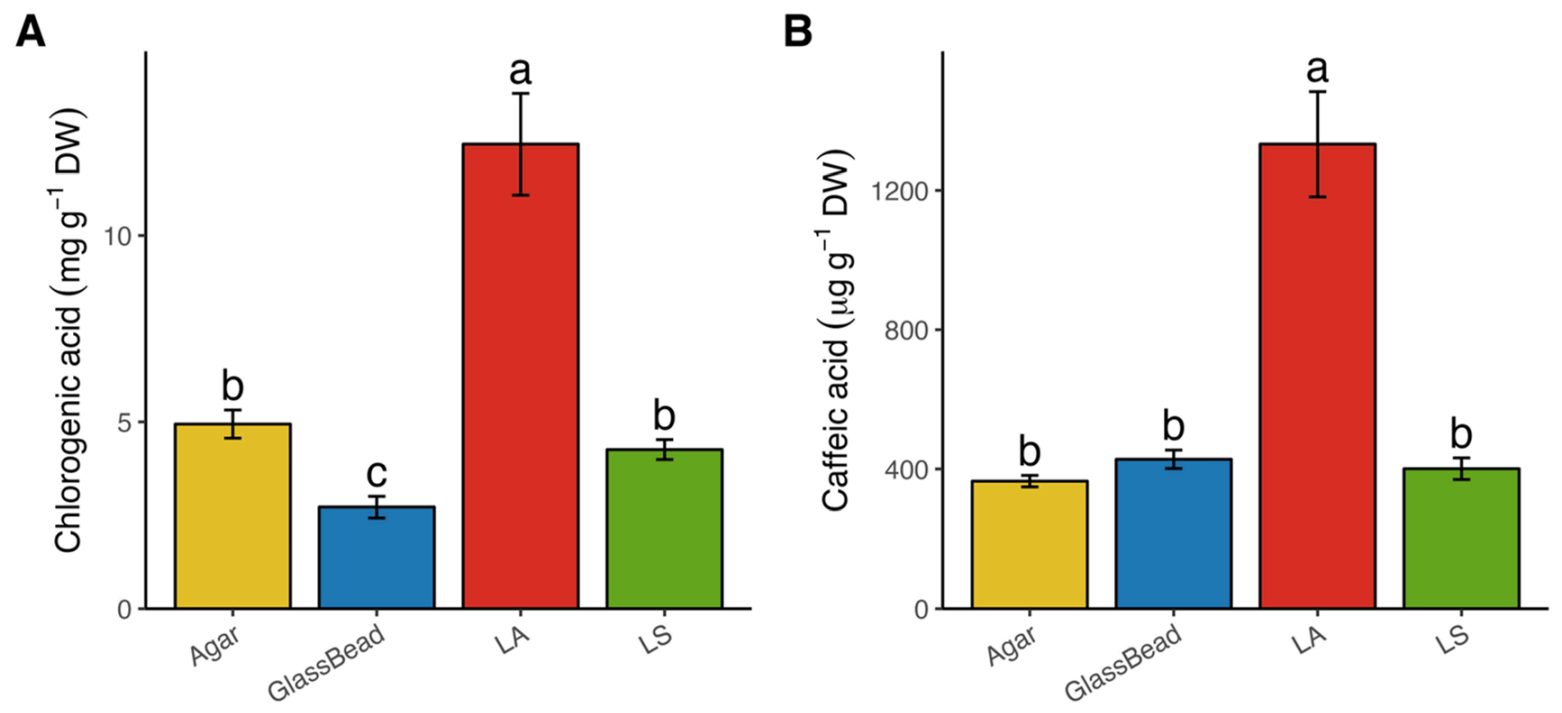
Chlorogenic acid (**A**) and caffeic acid (**B**) contents in aqueous extracts of four-week-old in vitro-grown stevia shoots cultured under agar-gelled, glass bead-supported, agitated liquid (LA), and static liquid (LS) culture systems. Data represent mean ± SD (*n* = 6). Different letters indicate significant differences according to Tukey’s HSD test (*p* < 0.05).

Shoots cultured under agitated liquid conditions accumulated the highest concentrations of chlorogenic acid (12.4 ± 1.4 mg g^−1^ DW) and caffeic acid (1.3 ± 0.2 mg g^−1^ DW), representing increases of approximately 2.5-fold and 3.6-fold, respectively, compared with agar-grown shoots (Figure 4).

### 2.3. Effects of Culture System on the Accumulation of Steviol Glycosides

Chromatograms recorded at 210 nm, especially those from shoots cultured on an agar-solidified medium or glass beads, showed two major peaks with retention times of about 11.3 min (peak 5) and 11.8 min (peak 6) (see Figure 3). Based on comparisons with authentic standards, these peaks were identified as rebaudioside A (Reb A) and stevioside (STV), respectively. Some other minor peaks were also detected, including signals at retention times of 16.0 and 16.7 min (peaks 7 and 8). Based on their chromatographic behavior and UV absorption characteristics, these peaks were tentatively classified as steviol glycosides.

**Figure 5:**
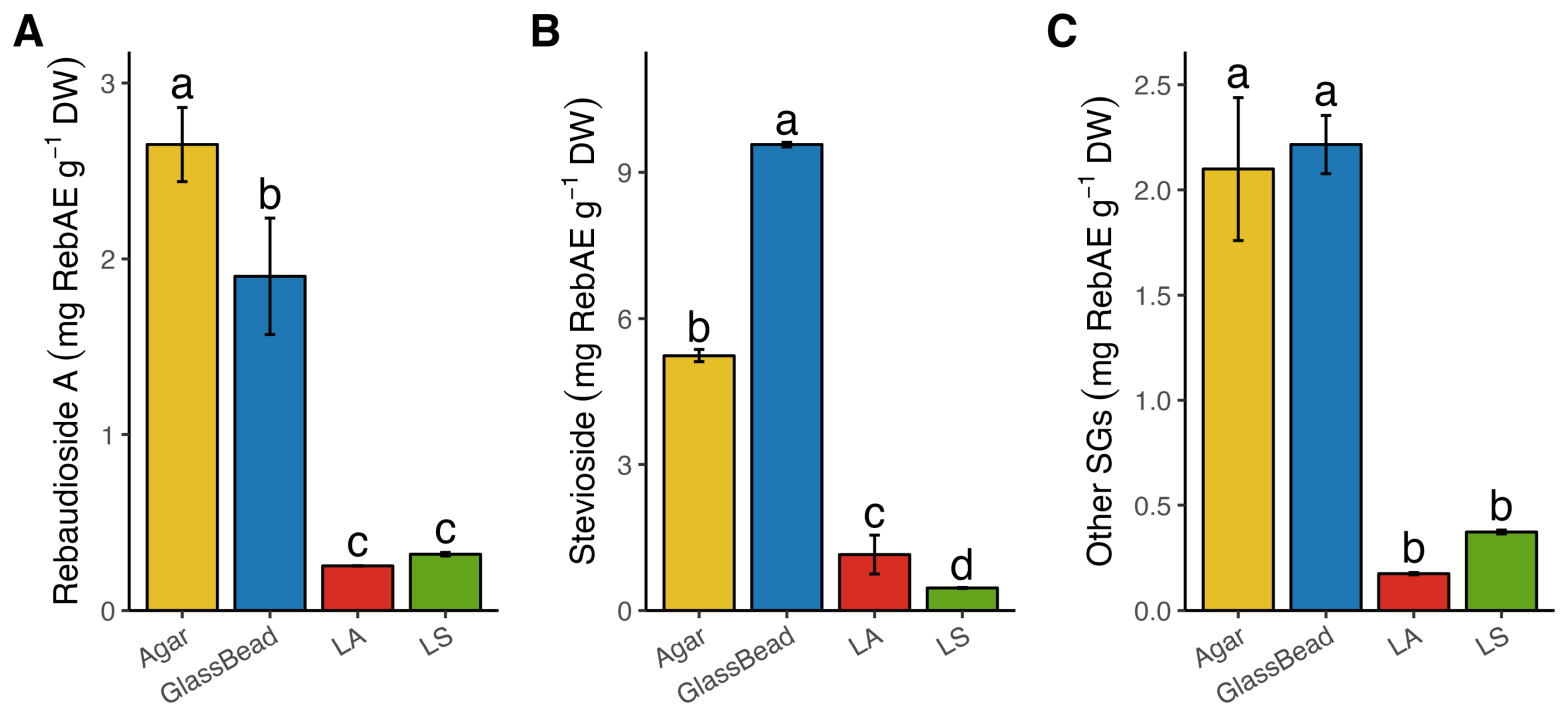
Rebaudioside A (**A**), stevioside (**B**), and total minor steviol glycosides (**C**) in four-week-old in vitro-grown stevia shoots cultured under agar-gelled, glass bead-supported, agitated liquid (LA), and static liquid (LS) culture systems. Data represent mean ± SD (*n* = 6). Different letters indicate significant differences according to Tukey’s HSD test (*p* < 0.05).

The concentrations of RebA, STV and the sum of the remaining steviol glycosides are presented in Figure 5. RebA accumulation was highest in shoots grown on agar-solidified medium (2.7 ± 0.2 mg g^−1^ DW), followed by those cultured on glass beads (1.9 ± 0.3 mg g^−1^ DW). In contrast, STV showed a markedly different pattern, reaching its highest concentration in glass bead cultures (9.6 ± 0.1 mg RebAE g^−1^ DW), substantially exceeding the levels detected in the other culture systems. The remaining steviol glycosides were present at similar concentrations in agar and glass bead cultures (approximately 2.1 mg RebAE g^−1^ DW), whereas considerably lower amounts were detected in liquid-grown shoots. Consequently, when all steviol glycosides were considered together, glass bead cultures exhibited the highest total accumulation (13.68 mg g^−1^ DW), followed by agar-solidified cultures (9.99 mg g^−1^ DW). In contrast, shoots grown in static and agitated liquid cultures accumulated only 1.2 and 1.6 mg g^−1^ DW of total steviol glycosides, respectively. A similar trend was observed for the RebA/STV ratio, which was highest in glass bead cultures (0.69 ± 0.01), intermediate in agar-solidified cultures (0.51 ± 0.04), and lowest in both liquid culture systems (approximately 0.22 ± 0.06).

### 2.4. Heatmap-Based Correlation and Principal Component Analysis

Taken together, the contrasting effects of the different culture systems on antioxidant capacity, phenolic compounds, and steviol glycoside accumulation suggested distinct metabolic response patterns. To further explore the relationships among variables and treatments, hierarchical clustering and principal component analyses were performed.

**Figure 6:**
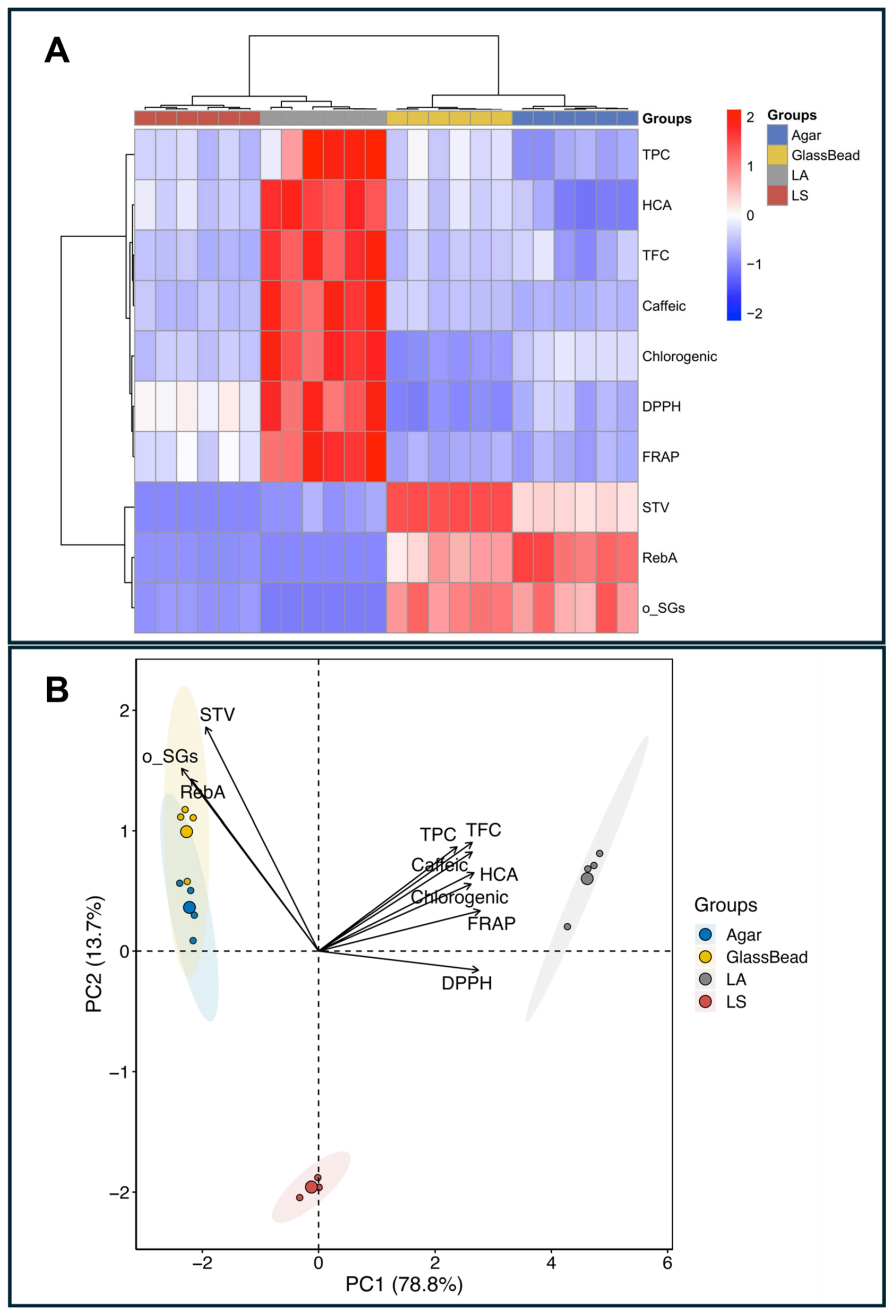
Hierarchical heatmap (**A**) and PCA biplot (**B**) based on the correlation matrix of biochemical parameters measured in in vitro-grown stevia shoots cultivated in four cultivation systems. In the hierarchical heatmap colors represent row-wise Z-scores of each measured parameter across all treatments: red indicates values above the mean for that parameter, and blue indicates values below the mean. Abbreviations: DPH, DPPH radical scavenging activity; FRAP, ferric reducing antioxidant power; o-SGs, other steviol glycosides; RebA, rebaudioside A; STV, stevioside; TFC, total flavonoid content; TPC, total phenolic content; LA, agitated-liquid culture; LS, static liquid culture.

Hierarchical clustering analysis revealed two main variable clusters (Figure 6A). The first cluster comprised steviol glycosides (Reb-A, STV, and other steviol glycosides), which were predominantly associated with agar-solidified and glass bead cultures. The second cluster grouped phenolic compounds (TPC, TFC, HCA, and chlorogenic and caffeic acids) together with TAC parameters (DPPH and FRAP), which were mainly associated with agitated liquid cultures and, to a lesser extent, with static liquid cultures. Treatment clustering separated solid-based (agar and glass bead) from liquid-based culture systems, reflecting distinct metabolic profiles. Pearson’s correlation analysis supported these relationships, revealing strong positive correlations among steviol glycosides and among phenolic compounds and antioxidant activity variables, whereas both groups were generally negatively correlated with each other (Supplementary Figure S2). Similarly, PCA confirmed these patterns (Figure 6B).

Similarly, PCA confirmed these patterns (Figure 6B). The first two principal components explained 92.9% of the total variance (PC1: 79.0%; PC2: 13.9%). PC1 separated liquid cultures (agitated and static) from solid-based systems. Phenolic compounds and antioxidant activity variables were positively associated with PC1 and clustered with liquid cultures, whereas steviol glycosides were negatively associated with PC1 and grouped with solid-based systems. PC2 explained a minor proportion of the variance and reflected residual variability among samples.

## 3. Discussion

This study shows that the in vitro culture system strongly influences the accumulation of steviol glycosides and phenolic compounds in *Stevia rebaudiana* shoots, highlighting the importance of culture conditions in modulating secondary metabolism. The total phenolic content (TPC) obtained in the present study was generally consistent with previous reports for *S. rebaudiana* grown under in vitro conditions. As expected, TPC values remained lower than those reported for greenhouse-grown plants, reflecting the increased accumulation of phenolic compounds typically observed under ex vitro conditions [28]. However, it is important to interpret comparisons among studies with caution because the methodology used for extraction can significantly influence the determination of TPC. Higher values have frequently been reported using hot-water extraction or subcritical water extraction of leaves from conventionally grown plants [29,30], whereas TPC values comparable to those obtained here have been described using hydroalcoholic extraction [31,32] or ultrasound-assisted aqueous extraction [33]. Collectively, these studies indicate that both cultivation conditions and extraction procedure—including solvent type, extraction temperature, and ultrasound-assisted techniques—strongly affect the recovery and quantification of phenolic compounds in *S. rebaudiana*.

Steviol glycosides showed a similar overall trend. The steviol glycoside concentrations obtained in this study were generally consistent with previous reports for *S. rebaudiana* grown in vitro. Rebaudioside A levels (1.90–2.65 mg g^−1^ DW) were comparable to those reported by [34,35], whereas stevioside accumulation (5.24–9.57 mg g^−1^ DW) exceeded values previously described for in vitro cultures. Nevertheless, both glycosides accumulated at substantially lower levels than those reported for greenhouse-grown plants, where stevioside and rebaudioside A may reach approximately 21 and 7 mg g^−1^ DW, respectively [36], in agreement with previous studies showing that in vitro-grown tissues generally contain lower steviol glycoside concentrations than ex vitro plants.

Plant tissue culture exposes explants to a unique combination of environmental and physiological conditions, including wounding, high sucrose concentrations, restricted gas exchange, high relative humidity, and exogenous plant growth regulators, which may induce physiological and oxidative stress responses compared with ex vitro growing conditions [37,38,39]. These conditions promote the production of reactive oxygen species (ROS), which act as important signaling molecules but, when produced in excess, can lead to oxidative stress and affect morphogenesis and plant development [40]. Plants counteract oxidative damage through a complex antioxidant defense network comprising both enzymatic and non-enzymatic components [41,42]. Among these, phenolic compounds represent one of the major classes of antioxidants, together with ascorbate, glutathione, carotenoids and tocopherols [41,42]. In addition to maintaining cellular redox homeostasis, these antioxidant systems also play a regulatory role in plant growth and development. Consequently, elevated antioxidant levels in plant tissues are widely regarded as indicators of both stress exposure and adaptive responses that contribute to tolerance against oxidative stress [43,44].

In the present study, total antioxidant capacity (TAC) was determined as an integrative indicator of the overall antioxidant status of the tissues [45]. Our results clearly showed that shoots cultured in liquid medium, particularly under continuous agitation, exhibited the highest TAC values, as demonstrated by both the DPPH radical scavenging and ferric reducing assays (Figure 1). A recent study revealed that higher levels of the lipid peroxidation marker malondialdehyde (MDA) were observed in spearmint shoots cultured under agitated liquid conditions compared to those grown in static liquid or agar-solidified media [21]. Therefore, the higher TAC seen in agitated liquid cultures likely reflects an increased accumulation of antioxidant metabolites aimed at maintaining cellular redox homeostasis and limiting oxidative damage. This interpretation aligns with prior reports indicating that abiotic stress or elicitation enhances the antioxidant capacity of stevia in vitro cultures, primarily due to increased phenolic compound accumulation [31,32,33,46]. We also observed an increase in the accumulation of total soluble phenolics, flavonoids, and hydroxycinnamic acids (Figure 2), as well as chlorogenic and caffeic acids (Figure 3A and Figure 4), in stevia shoots cultured under agitated conditions, compared to other culture systems. Furthermore, hierarchical clustering and PCA revealed a close association between phenolic variables and TAC (Figure 6), which supports the contribution of these compounds to the antioxidant capacity of aqueous stevia extracts. Stevia leaves are recognized as an important source of caffeoylquinic acids (CQAs), including chlorogenic acid and its derivatives. These compounds are considered major contributors to the antioxidant activity of stevia extracts [9,47,48,49,50]. Consistent with this, our HPLC analysis showed increased accumulation of chlorogenic and caffeic acids in shoots cultured under agitation. This parallels the higher TAC values and supports the contribution of these hydroxycinnamates to the antioxidant response induced by this culture system.

In contrast to the phenolic compounds, which accumulated under agitated liquid conditions, steviol glycosides reached their highest levels in the solid-supported culture systems (Figure 3B). Steviol glycosides (SGs), the diterpenoid sweeteners responsible for the characteristic sweetness of *Stevia rebaudiana*, comprise more than 60 compounds, with stevioside (STV) and rebaudioside A (Reb A) being the predominant constituents of the leaves [3,4]. In our study, Reb A reached its highest concentration in shoots cultured on agar-solidified medium, whereas STV accumulated predominantly in glass bead cultures (Figure 5). Overall, both solid-supported culture systems promoted greater SGs accumulation than the liquid culture systems. This trend is consistent with the findings of [51], who likewise reported higher STV and RebA contents in leaves of stevia shoots cultured on agar-solidified MS medium than in liquid cultures. Similar results were reported by [52], who found that RebA accumulated 27-fold more in the leaves of shoots cultured on agar than in those from agitated liquid cultures. None of the aforementioned studies analyzed the TAC or levels of phenolic compounds [51,52], although [52] determined the activity of some antioxidant enzymes and claimed that the increase in RebA was associated with higher levels of oxidative stress. While the results are aligned, it is evident that further studies are necessary to address the apparent discrepancy between the conclusions of our work and those reported in [52].

In any case, most published studies show that SGs contribute very little to the antioxidant capacity measured in stevia tissues [53,54]. While some authors have reported that purified SGs exhibit antioxidant properties [55], the lack of correlation between antioxidant capacity and SG levels suggests that SGs are not primary players in defense mechanisms against oxidative stress. This has been confirmed by subjecting stevia plants grown in vitro and in greenhouses to stressful conditions. For instance, saline treatments typically result in increased levels of antioxidant compounds (both phenolic and non-phenolic) under both growing conditions, while SG levels tend to remain unchanged or decrease [32,56]. Despite the absence of significant alterations in SG levels as indicated by [32], an augmentation in the expression levels of select pivotal genes within the SG biosynthetic pathway was observed by those authors. This finding aligns with the modest elevations in SG levels reported by several researchers at relatively low salt concentrations [56,57]. Similar results have been obtained in glass bead cultures of stevia shoots exposed to increasing concentrations of methanol—modeling the operation of the pectin/pectin methyl esterase/methanol system—yielding substantial increases in SG levels at low concentrations (0.1%) and reductions at higher concentrations, accompanied by an augmentation in phenolic compound content [33]. These observations support the hypothesis that there exist on/off thresholds for the expression of distinct biosynthetic pathways, which are stimulated by varying stress levels.

The contrasting responses of phenolic compounds and SGs suggest that these metabolic pathways are differentially regulated under the tested culture conditions. Whereas agitation favored the accumulation of antioxidant phenolics, solid-supported cultures promoted the biosynthesis of the diterpenoid sweeteners, indicating that the conditions maximizing antioxidant metabolism do not necessarily coincide with those favoring SG production. From an applied perspective, this makes it possible to select the in vitro culture system to be used with stevia to increase the production of a specific type of metabolites.

## 4. Materials and Methods

### 4.1. Plant Materials and Growth Conditions

Single-node nodal segments were excised from in vitro cultures of *S. rebaudiana* Bertoni cv. Criolla previously established in our laboratory [32,33,58]. Stock cultures were maintained on a modified Murashige & Skoog (MS) medium [59], containing half-strength macronutrients (MS/2), supplemented with casein hydrolysate (250 mg L^−1^), sucrose (30 g L^−1^) and agar (8 g L^−1^). Cultures were grown under a 12-h photoperiod with a photosynthetic photon flux density of 75 μmol m^−2^ s^−1^, with temperatures of 25 °C during the light period and 18 °C during the dark period. Shoots were subcultures every four weeks.

**Figure 7:**
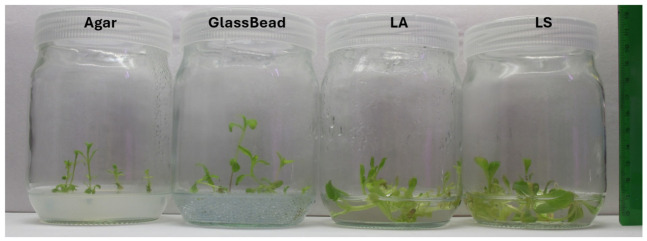
Representative photographs illustrating the four culture systems used in this study and the morphology of in vitro-grown stevia shoots grown under each culture system for 11 days. All culture vessels contained 25 mL of MS/2-based nutrient medium. From left to right: Agar-gelled medium, glass bead-supported liquid medium, agitated liquid medium (LA), and static liquid medium (LS). LA cultures were maintained on an orbital shaker at 60 rpm throughout the cultivation period.

For the experiment, four-week-old in vitro-grown stevia shoots were divided into single-nodal explants. Four explants were placed in 580-mL glass jars containing 25 mL of MS/2 salt medium supplemented with casein hydrolysate and sucrose as described above. Four culture systems were evaluated: (i) agar-solidified medium (8 g L^−1^), (ii) glass bead-supported cultures consisting of 80 g of sterile glass beads (2 mm in diameter) placed over 25 mL of liquid MS/2 medium per vessel, (iii) agitated liquid cultures, and (iv) static liquid cultures (Figure 7). Agitated cultures were maintained on an orbital shaker (Orbitron INFORS HT, Bottmingen, Switzerland) at 60 rpm throughout the cultivation period.

After 30 d, the shoots (including stems and leaves) from each culture system (agar-solidified, glass bead-supported, static liquid, and agitated liquid cultures) were harvested, oven-dried at 60 °C for 72 h (Incudigit-TFT, J.P. Selecta, Abrera, Spain), and ground into a fine powder. The powdered samples were transferred to airtight tubes and stored at −20 °C until analysis.

The experiment was performed twice independently at different times. In each experimental run, each treatment consisted of three biological replicates (culture vessels), with each biological replicate containing four shoots. All cultures were maintained under the same temperature and light conditions described above.

### 4.2. Plant Extract Preparation

Dried powdered plant material (200 mg) was extracted with 4 mL of Milli-Q water. Samples were vortexed, sonicated (37 kHz; FB-15049, Fisher Scientific, Pittsburgh, PA, USA) for 30 min at 80 °C, and centrifuged at 15,000× *g* for 10 min at 4 °C. The resulting supernatants were collected, aliquoted, and stored at −80 °C until analysis.

### 4.3. Determination of the Total Antioxidant Capacity

Total antioxidant capacity was assessed using the 2,2-diphenyl-1-picrylhydrazyl radical (DPPH) radical scavenging assay and the ferric reducing/antioxidant power (FRAP) method, as previously described [60,61]. DPPH activity was expressed as µmol cholorogenic acid equivalent (CAE) per gram of dry weight (DW), whereas FRAP was expressed as µmol Fe(II) per gram DW.

### 4.4. Determination of Total Phenolic, Flavonoid and Hydroxycinnamic Acid Contents

The total soluble phenolic content (TPC), total soluble flavonoid content (TFC) and total soluble hydroxycinnamic acid content (HCA) were determined according to previously reported methods [33,60,61,62]. Briefly, TPC was quantified using the Folin–Ciocalteu assay, with chlorogenic acid (0–3000 µM) as the standard, and expressed as µmol chlorogenic acid equivalents (CAE) per gram of DW. TFC was determined using the aluminum chloride method with rutin (0–2000 µM) as the standard and expressed as µmol rutin equivalents (RE) per gram DW. HCA content was determined using the Arnow’s reagent [10% (*w*/*v*) of sodium molybdate and 10% (*w*/*v*) sodium nitrite], with chlorogenic acid (0–3000 µM) as the reference standard, and expressed as µmol CAE per gram of DW.

### 4.5. HPLC Analysis

Phenolic compounds and steviol glycosides in aqueous extracts of *S. rebaudiana* in vitro shoots were identified and quantified by reversed-phase high-performance liquid chromatography (RP-HPLC) according to [63]. Briefly, 10 µL of each extract was injected into a Phenomenex Luna C18 reversed-phase column (250 mm × 4 mm, 5 μm particle size) coupled to a Waters Alliance 2695 HPLC system equipped with Waters 2996 photodiode array detector and controlled using Empower Pro software 2002 (Waters Corporation, Milford, MA, USA). The mobile phase consisted of acetonitrile and 10 mM phosphate buffer (pH 2.6) at a constant ratio of 32:68 (*v*/*v*). Separation was performed under isocratic elution at a flow rate of 1 mL min^−1^. Compounds were identified by comparing their retention times and UV spectra with those of authentic standards, including caffeic acid, chlorogenic acid, gallic acid, quercetin and rebaudioside A.

Chromatograms were recorded at 210 nm (steviol glycosides), 280 nm (total phenols), 325 nm (hydroxycinnamic acid derivatives), and 360 nm (flavonoids). Quantification was performed using the external standard method, and results were expressed as μmol equivalents of the corresponding standard per gram DW.

### 4.6. Statistical Analysis

Data are presented as mean ± standard deviation (SD). Since the two independent experiments showed similar treatment responses, the data were combined for the final statistical analysis. This resulted in six biological replicates per treatment. Differences among treatments were assessed by one-way analysis of variance (ANOVA), followed by Tukey’s honestly significant difference (HSD) post hoc test. Statistical significance was established at *p* < 0.05. Statistical analyses were performed using IBM SPSS Statistics version 28.0 (IBM Corp., Armonk, NY, USA).

Heatmaps were generated using the *pheatmap* package in R. Data were row-scaled (z-score normalization), and hierarchical clustering was performed using Pearson correlation distance and complete linkage.

Principal component analysis (PCA) was performed in R (version 4.3.1; https://www.R-project.org/ (accessed on 6 July 2026)) using the *FactoMineR* package on centered and scaled data. PCA biplots were generated using the *factoextra* package.

Pearson correlation coefficients were calculated in R using the base function *cor()*. The resulting correlation matrix was visualized as a color-coded correlation heatmap using the *corrplot* package.

## 5. Conclusions

This study shows that the configuration of an in vitro culture system plays a key role in modulating the production of metabolites in *Stevia rebaudiana* shoots. Agitated liquid cultures increased the accumulation of phenolic compounds and antioxidant capacity, while the glass bead system favored the production of steviol glycosides. These results confirm that different culture environments can selectively influence metabolic pathways.

From a biotechnological perspective, selecting an appropriate culture system is a practical strategy for directing the production of specific target compounds. This approach can contribute to optimizing in vitro systems for large-scale production of stevia-derived metabolites.

Future studies should focus on scaling up these systems and elucidating the physiological and molecular mechanisms underlying the observed differences in metabolite accumulation.

## Data Availability

The original contributions presented in this study are included in the article/Supplementary Material. Further inquiries can be directed to the corresponding author.

## Supplementary Materials

The following supporting information can be downloaded at https://www.mdpi.com/article/10.3390/plants15152326/s1, Figure S1: Representative HPLC chromatograms obtained at 325 nm (A) and 210 nm (B) from aqueous extracts of in vitro stevia shoots cultured under agar-gelled, glass bead-supported, agitated liquid (LA), and static liquid (LS) culture systems; Figure S2: Pearson correlation matrix among biochemical parameters measured in *Stevia rebaudiana* shoots cultured under four in vitro cultivation systems.

## Abbreviations

The following abbreviations are used in this manuscript:

HCA	Total soluble hydroxycinnamic acid content
RebA	Rebaudioside A
SGs	Steviol glycosides
TAC	Total antioxidant capacity
TFC	Total soluble flavonoid content
TPC	Total soluble phenolic content
