# Supplementary material for: Influence of the In Vitro Culture System on the Preferential Accumulation of Bioactive Compounds in Stevia rebaudiana Shoots
Source: Plants (Basel). 2026 Jul 29;15(15):2326. doi: 10.3390/plants15152326 (PMC13467675; doi:10.3390/plants15152326)

Supplementary material

Supplementary Figure S1: Representative HPLC chromatograms obtained at 325 nm (A) and 210 nm (B) from aqueous extracts of in vitro stevia shoots cultured under agar-gelled, glass bead-supported, agitated liquid (LA), and static liquid (LS) culture systems. Insets show enlarged views of the chromatographic regions where the target compounds eluted (approximately 0-5 min in A and 10-20 min in B). Peak 1, chlorogenic acid; peak 2, caffeic acid; peaks 3 and 4, unidentified hydroxycinnamic acid derivatives; peak 5, rebaudioside A (RebA); peak 6, stevioside (STV); peaks 7 and 8, unidentified steviol glycosides (SGs).

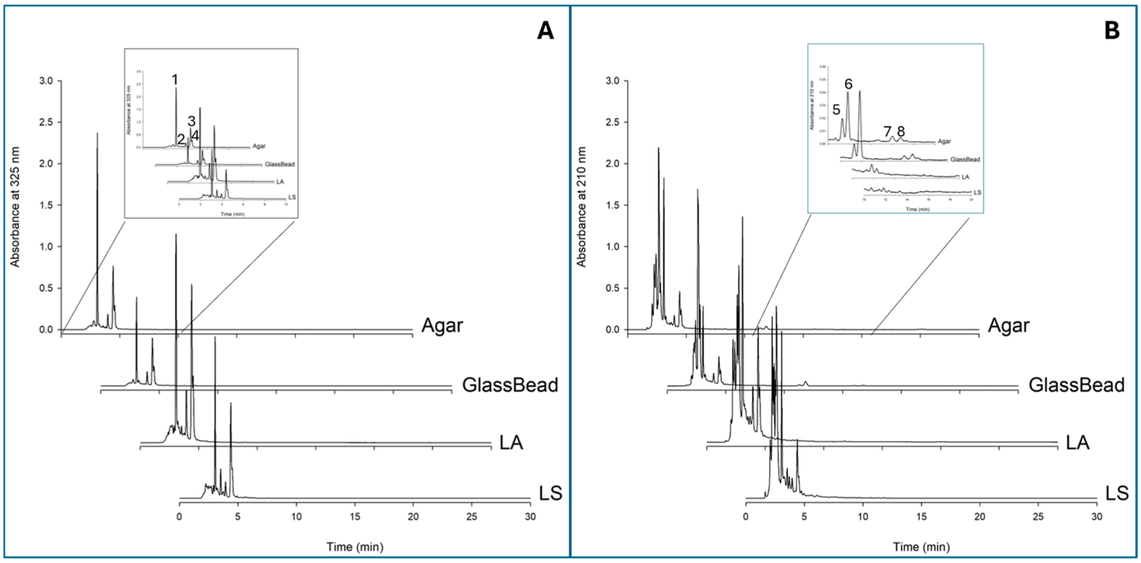

Supplementary Figure S2. Pearson correlation matrix among biochemical parameters measured in *Stevia rebaudiana* shoots cultured under four in vitro cultivation systems. Cells show Pearson correlation coefficients (r). Blue and red indicate positive and negative correlations, respectively, and color intensity reflects the strength of the correlation. Only statistically significant correlations ($p < 0.05$) are displayed.

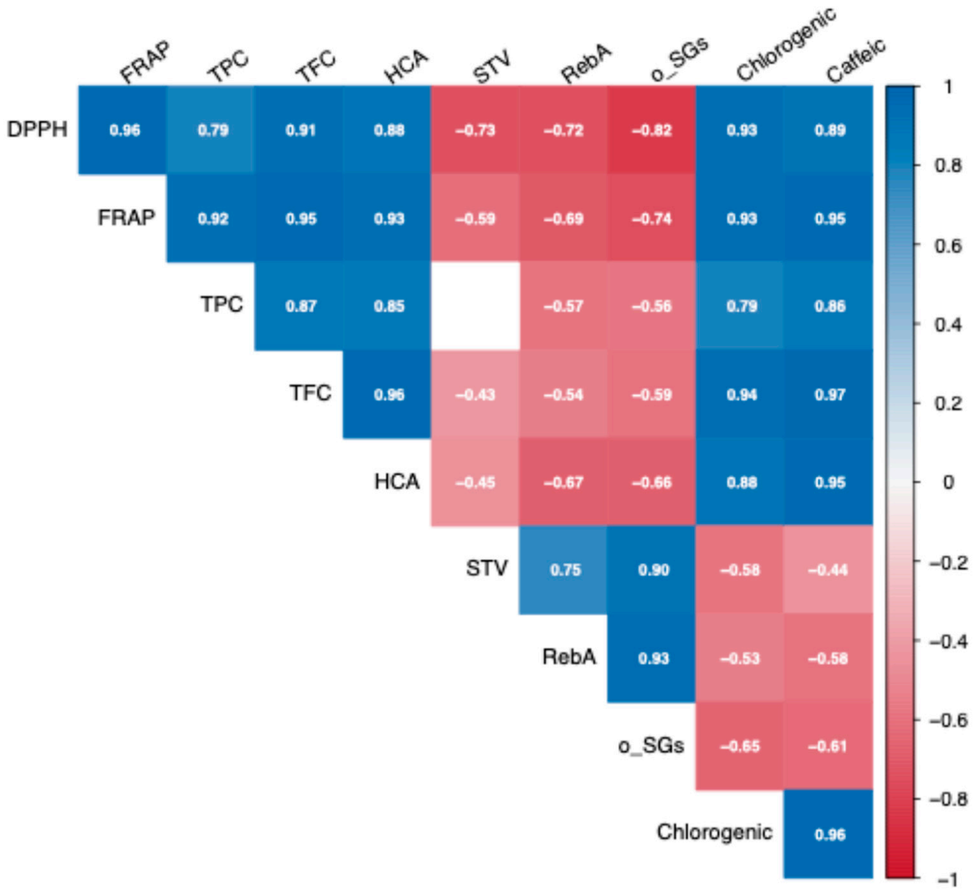

Supplement: Supplementary file 1 [file plants-15-02326-s001.zip › plants-4452360-supplementary.pdf]
